# Kinematic and Gait Similarities between Crawling Human Infants and Other Quadruped Mammals

**DOI:** 10.3389/fneur.2015.00017

**Published:** 2015-02-09

**Authors:** Ludovic Righetti, Anna Nylén, Kerstin Rosander, Auke Jan Ijspeert

**Affiliations:** ^1^Autonomous Motion Department, Max-Planck Institute for Intelligent Systems, Tübingen, Germany; ^2^Uppsala Child and Baby Lab, Department of Psychology, Uppsala University, Uppsala, Sweden; ^3^Biorobotics Laboratory, Interfaculty Institute of Bioengineering, School of Engineering, École Polytechnique Fédérale de Lausanne, Lausanne, Switzerland

**Keywords:** infant locomotion, crawling, gait, kinematics, quadruped locomotion

## Abstract

Crawling on hands and knees is an early pattern of human infant locomotion, which offers an interesting way of studying quadrupedalism in one of its simplest form. We investigate how crawling human infants compare to other quadruped mammals, especially primates. We present quantitative data on both the gait and kinematics of seven 10-month-old crawling infants. Body movements were measured with an optoelectronic system giving precise data on 3-dimensional limb movements. Crawling on hands and knees is very similar to the locomotion of non-human primates in terms of the quite protracted arm at touch-down, the coordination between the spine movements in the lateral plane and the limbs, the relatively extended limbs during locomotion and the strong correlation between stance duration and speed of locomotion. However, there are important differences compared to primates, such as the choice of a lateral-sequence walking gait, which is similar to most non-primate mammals and the relatively stiff elbows during stance as opposed to the quite compliant gaits of primates. These finding raise the question of the role of both the mechanical structure of the body and neural control on the determination of these characteristics.

## Introduction

Despite the vast variety of quadruped mammals, their locomotion behaviors have a lot in common in terms of gait, kinematics, and neural control (1–5). Studying these common aspects seems therefore fundamental to the understanding of quadruped locomotion, from the mechanical determinants imposed by physical constraints to the neural control of locomotion.

For example, most mammals use very similar gaits that change for different speeds. Symmetrical gaits such as walk, trot, and pace are used at slow and moderate speeds (6, 7) and asymmetrical gaits are preferred at high speeds (8). In vertebrate quadruped locomotion, the duration of stance phase is directly related to the locomotion speed while the swing phase stays almost constant for most speeds (3, 5). Mammals also share many kinematic similarities. They have similar periods of flexion and extension of the shoulder and hip, two period flexion/extension of the more peripheral joints, and lateral and sagittal movement of the spine (1, 2, 5, 9).

However, non-human primate locomotion can be distinguished from other quadrupeds in several important ways. While most mammals use a lateral-sequence walking gait [swing sequence: left-hind (Lh), left-front (Lf), right-hind (Rh), and right-front (Rf)], primates use mainly a diagonal sequence gait LhRfRhLf (7, 10). Even if lateral-sequence walking can be observed, the diagonal sequence is always the dominant one (11). It has been suggested that this gait was first evolved for fine branch locomotion (12). Primates also have a more protracted arm at touch-down (over 90° relative to horizontal body plane) than other mammals (13), a quite compliant gait characterized by important elbow yields during stance, longer steps and longer contact times, and relatively extended limbs during stance (14). However, the exact reason for these differences remains controversial.

Humans only exhibit quadruped locomotion behaviors during their infancy or in some pathological cases during adulthood, for example, persons afflicted by the Uner Tan Syndrome (15). The study of human infant crawling can help understand fundamental invariants of quadrupedalism across mammals. It is also a step toward understanding more pathological forms of quadrupedalism during adulthood. Infants have a crawling posture that is different from other quadrupeds and not optimized for quadrupedalism. Crawling infants have only two functional limb segments for the fore limb (arm and forearm) and one for the hind limb (since knees are on the ground during stance) and the scapula is not aligned with the shoulder. Consequently, the study of infant crawling is the study of quadrupedalism in mechanically more constrained and likely non-optimal form. All the gait characteristics of infants common to other mammals would suggest that these are independent of the functional limb geometry of the quadruped. It would then emphasize the importance of either the neural control or the constraints imposed by quadrupedalism (but independent from specific limb geometry) in the emergence of these common characteristics. Primate locomotion differs from other quadrupeds in several aspects (12, 13, 16) and it is not known how it is related to young human infant’s locomotion. Indeed, although Hildebrand (7) gives a direct comparison of the interlimb coordination between primates and infants and shows that infants use lateral-sequence walks that differ from primates but are similar to most other quadruped mammals, there is no data for the specific kinematics of the limbs.

The development of crawling is similar to other motor skills, as sitting, cruising, and walking. This behavior is a result of improved posture, neuromuscular control, and experience. The importance of postural and neuromuscular control is reflected by the fact that at the very onset of crawling, infants use hands and knees immediately in an optimal way. The right hand starts the locomotion (17). However, the crawling technique is highly variable, in other respects depending on clothing (18) or friction (17). The infant prefers a crawling pattern that fits the environmental conditions. In the past, several studies have mapped up crawling stages and crawling patterns (18, 19), and the refined neural control with age (19–21). These characteristics show a high variability with age and with environmental conditions. More recent studies have emphasized the strong role of experience. For example, cross-cultural studies show that training before the crawling onset gives an earlier start (22). It has been shown that at the onset of locomotion like cruising and walking, the postural systems involved in the perception–action loop are not perfect yet. In cruising, (7–12 months) the infant wobbles, which is decreased with experience (23). Similarly, Adolph (24) has suggested a sway model for locomotion.

Despite the importance of infant crawling there are very few studies of the biomechanical properties of this mode of locomotion. Infants start crawling at around 9 months (25) and continue until they start walking. They may have very different strategies for crawling (17), using either three or four limbs, the belly touching the ground or not, with different types of limb coordination, but their most common gait is a walking trot with alternating locomotion of the ipsilateral hand and contralateral knee in sequence (hereafter called “the standard crawling gait”). It is the gait that we propose to study in more details.

Burnside (18) and Hildebrand (7) were the first to report quantitative data on interlimb coordination during crawling on hands and knees. The coordination pattern is between a walking trot and a lateral-sequence walk. Hildebrand also reported a difference in the durations of stance between hands and knees (hand stance is between 120 and 130% longer than for the knees), which is high compared to other quadrupeds. He also remarked that the concept of gait for human infants is less meaningful than for other mammals due to the unsteadiness of infant locomotion. Recently, Patrick et al. (26) tested 26 human infants and 7 adults in various conditions (treadmill and normal ground). They also observed that the crawling pattern was mainly restricted to a lateral-sequence walk and symmetric running patterns were never seen. They reported gait transitions in infants crawling but these transitions were smooth variations of the ipsilateral phase lag (between the fore and hindlimbs), which can be related to the unsteadiness nature of infant crawling.

There are almost no studies of the kinematics of infant crawling. Although Mucino et al. (27) and Niemitz (28) reported kinematic data on infants for the different limbs, their experiments were only done on one and two infants, respectively, and no extensive analysis of these data or comparisons with other mammals was done. Moreover, to the best of our knowledge, there are no quantitative studies available about the crawling gait in human infants that combine both gait analysis and kinematic data as compared to other mammals.

In this study, we provide an analysis of how infant crawling gait differs from other quadrupeds, in particular non-human primates, looking at basic limb kinematics and interlimb coordination. The paper gives a detailed description of the standard crawling gait of human infants and compares its characteristics with other quadrupeds and especially primates. These comparisons are done in terms of: (1) the basic limb kinematics, (2) the relation between speed of locomotion and swing/stance durations, (3) the preferred footfall sequences, and (4) the coordination between the limbs and the spine. Furthermore, we asked how development improves the crawling patterns by measuring three infants twice.

## Materials and Methods

### Subjects

Nine healthy infants, 9–11 months old, practicing the standard crawling gait participated in the study (see Table 1). Three of them were measured twice (A, B, and C in Table 1). Two subjects were excluded due to incomplete data collection (not shown in Table 1). On most trials these two infants only made one step and stopped. The following discusses the results obtained with seven infants, three of them measured twice. They were all healthy infants (four boys, three girls), full term with normal birth weight, and according to parents without any complications or illnesses during the neonatal period.

**Table 1:** **Data of birth, experience of crawling^a^, number of complete steady crawling cycles that were extracted from the experiments for each limb and body mass the day of the experiment for the seven infants^b^**.

Infant	Age (days)	Experience (days)	Numbers of complete cycles				Body mass (kg)
			Left arm	Right arm	Left leg	Right leg	
A	253/296	28/71	17/6	16/10	13/2	16/7	7/7.5
B	273/301	21/49	12/5	10/8	10/6	11/7	11/12
C	286/332	15/61	16/5	15/6	8/6	10/6	9.5/10
D	290	59	6	5	5	5	9
E	304	39	12	13	11	13	11
F	319	89	1	5	4	2	10.5
G	290	21	13	7	10	3	10

*^a^The number of days since estimated start of crawling*.

*^b^Note that A., Al., and E. were seen two times*.

### Procedure

The study was performed in accordance with the ethical standards specified in the 1964 Declaration of Helsinki and was approved by the ethics committee of Uppsala University. When the parents came to the lab they were informed about the experiment and signed a consent form. They obtained a gift certificate of value 10 € for participation. The parents undressed the infants and 18 small markers (diameter 4 or 8 mm, respectively) of reflective material were attached to the skin on the joints or close to them. Three markers were put on the spine (neck, thoracal, and lumbar). The subjects wore a hat on which three markers were attached (one midsagittal and two coronal). The markers on the wrists and knees were glued to a velcroband. This gave stability to the critical parts that were close to the floor during locomotion. One disadvantage was that the knee markers were just above the joint. The remaining markers (elbows, shoulders, hips, and feet) were attached with double-sided tape used for skin electrodes. When all 18 markers were properly attached the infant was encouraged to crawl on a rug (polypropylene, size 230 cm × 170 cm) placed on the floor. The parent and one experimenter were sitting on the floor on opposite sides of the rug using attractive toys to catch the infant’s attention. A second experimenter sitting close to the rug handled the measurements and observed the infant’s behavior. Each trial was video-recorded in synchrony with the measurements.

### Measurements

A motion capture system, “Proreflex (Qualisys),” was used to measure the movements in 3-D space. Data were collected at 240 Hz for 12 s periods sampled with external pre-triggering. In close synchrony with the measurement sessions, a video camera monitored the infant during the trials. Five Qualisys cameras were used, two were placed on ceiling stands and three were placed on the floor so that the crawling area was covered. When the infant showed intention to start crawling, the second experimenter started the measurement. Usually between 20 and 40 trials per infant were recorded. Only trials containing at least one complete gait cycle were taken into account. Valid trials contained on average three complete cycles (see below for details).

### Data processing

For each measurement, the markers were identified and their positions translated to Euclidian coordinates (Qualisys software) with an accuracy of 0.5 mm. Data were then processed (Matlab, Mathworks) in order to interpolate for missing data over small time intervals (<200 ms) and to remove high frequency noise. First, a piecewise cubic Hermite interpolation is used for the missing data. Note that no extrapolation of the data was done. Second, a locally weighted scatter plot smoothing is used to remove noise. It used least squares linear polynomial fitting with 20 data points for each local smooth calculation (span of 83 ms).

#### Swing-stance measurement

In order to have comparable data for steady state crawling, only the crawling sequences in which the infant was crawling straight toward a goal without stopping to do something else were selected. Only complete gait cycle sequences were taken into account. Therefore, a stance phase was always measured between two swing phases. Table 1 shows the final number of complete cycles obtained for each infant that were used in the analysis below. The swing phase of the arms/legs was defined as the phase during which the hands/knees were moving forward.

**Figure 1:**
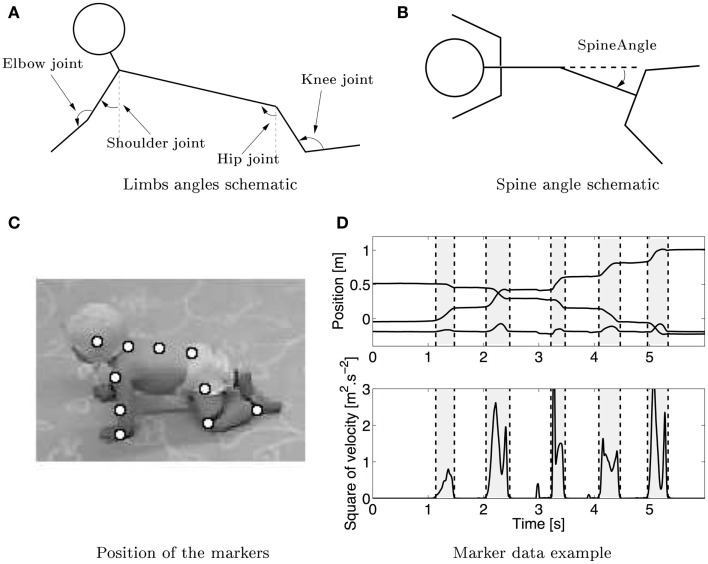
**(A)** Schematic (not based on real data) of the measured joint angles in sagittal plane. The shoulder, elbow, hip, and knee joints are measured. The arrows show the positive angles. The shoulder and hip joint were measured relative to the vertical. **(B)** Schematic of the spine angle made by the three markers on the spine measured in the lateral plane, the arrow shows the positive direction of the angle. **(C)** Snapshot of a crawling infant together with the position of the markers that were used to calculate the different angles. **(D)** Typical trajectory of the hand in x–y–z directions (upper graph, the lower line is the z direction, while the middle and upper one are respectively the y and x directions) during crawling with the corresponding squared velocity profile. Vertical dashed lines separate swing (gray) and stance (white) phases.

The onset of the swing phase is found by computing the squared time derivative of the positions of the hands and knees. A threshold is used to decide when the limbs are moving and is defined as the value above the maximum value found during the middle of the stance phase averaged over all the stance phases (Figure 1). Video recordings are systematically used to check measures consistency and to correct them when necessary (i.e., to exclude wrong swing measurements).

#### Kinematic measures

Five different degrees of freedom (DOFs) were chosen for study: the angle of the shoulder in the sagittal plane, the elbow of both arms, the hip in the sagittal plane, the knee for both legs, and the spine angle in the horizontal plane. Figure 1 shows the different DOF, the shoulder, and hip joints have an angle of 0 when they are vertical. The elbow and knee joints are taken to be 0 when they are completely flexed (note that it is physically impossible). These measurements are compatible with other kinematic studies [e.g., Larson et al. (29)] and therefore enable comparison with previous studies.

The median movement of the different DOFs is computed. For each infant, the swing and stance phases are scaled separately for each trajectory by means of local linear interpolation. The median value of all the data set for each point in time is then calculated. The duration of the swing is set to 40% of a complete cycle and the stance to 60%, which correspond to a typical value for the crawling gait. This scaling allows comparison of limb kinematics between different crawling gaits with different stance and swing durations.

For the case of the DOF of the spine, this median movement is rescaled in the same way as for the joints, except that it is centered at 0°. The different phases of the movement are defined in two ways. In the first case, the phases are defined by the swing phase of the left arm, the period when the four limbs were on the ground, the swing of the right arm, and the period when the four limbs are on the ground again. In the second case, the phases are defined with reference to the swing of the legs (swing of right leg, complete support, swing of left leg, and complete support). These two representations allow studying the coordination of the spine with the four limbs. A positive value for the spine angle means that the spine is folding in the left direction (Figure 1).

#### Statistical measures

The median and interquartile range estimators are used instead of the mean and standard deviation, as the former estimators are more robust against noise and outlier values (30). All the results are evaluated using non-parametric tests that do not assume a Gaussian distribution of the data. Whenever needed, the Spearman correlation tests and Wilcoxon rank sum tests are used. The level of statistical significance is set to 5%.

## Results

### Gait analysis

#### Swing and stance durations

**Figure 2:**
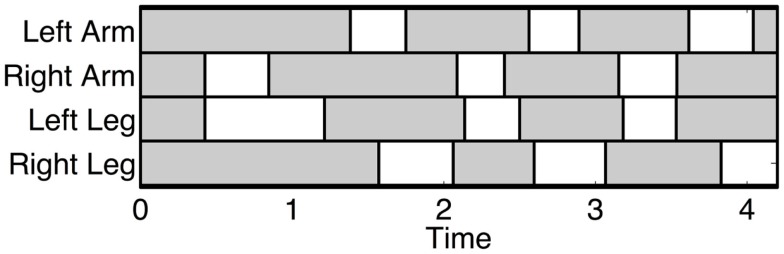
**Typical footfall sequence (real data) of the infant standard crawling gait**. The dashed boxes show the stance phases and the white ones the swing phases. In this case, the infant starts to crawl when the right arm swing first. The graph shows the long stance durations, the walking trot gait, and the fact that the arms swing slightly before the diagonal limbs.

The crawling gait is characterized by almost synchronous movements between the ipsilateral arm and the contralateral leg. The ipsilateral arm is roughly half a period out of phase with the contralateral arm. The swing phases of the ipsilateral limbs never overlap. Figure 2 shows the typical footfall sequence of this gait.

The gait characteristics are quantified by computing the duty factor, diagonality, and symmetry of the gait as defined by Hildebrand (see in Ref. (6) for more details on gait characteristics). The percentage of the cycle period by which the left arm (resp. left leg) footfall precedes the right arm (resp. right leg) footfall is always around 50%, indicating a symmetric gait. The duty factor (stance period of the hind legs as a percentage of stride duration) is comprised between 50 and 70% and the diagonality (percentage of the cycle period by which the left-hind footfall precedes the left fore footfall) between 33 and 40%. The standard crawling gait therefore corresponds to a gait between a walking trot and a lateral-sequence diagonal couplets walk^1^. Experimental data also show a discrepancy between the fore and hind limbs duty factor, where the fore limbs duty factor is on average 8% higher than the hind limbs one. This difference is statistically significant for 5 out of 7 infants (*p* < 0.05).

The median duration of the arm swing is between 300 and 446 ms for the seven infants, the median duration of the leg swing is between 354 and 554 ms. The variability of the swing phase (the ratio of the interquartile range of the swing duration to its median duration for each infant) has a median of 16%. Compared to this, the median duration of the stance of the arms for the different infants varies between 367 and 1035 ms, and the median duration of the stance of the legs between 373 and 975 ms. The variability of the stance duration (ratio of the interquartile range of the stance duration with its median duration for one infant) has a median of 28%. Interestingly, the duration of the swing phase has small variability both within each measured infant and between all the infants. However, the variability of the stance duration is much more pronounced for each infant and especially between infants.

#### Relation between speed and cycle duration

**Figure 3:**
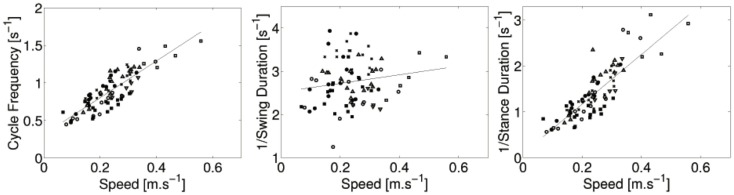
**Cycle frequency, 1/stance duration, and 1/swing duration as a function of the speed of locomotion for all subjects:** ● for infant A, ○ for B, ♦ for C, ◇ for D, ■ for E, □ for F, and × for G. Regression lines are also showed. The strong linear correlation between locomotion speed and cycle frequency (resp. 1/stance duration) is noticeable.

The crawling velocity is estimated from the positions of the markers located on the spine. Figure 3 shows the cycle frequency, 1/stance duration, and 1/swing duration as a function of the velocity. There is a strong linear relation between the frequency of the cycle and the locomotion speed (*r* = 0.86, *p* < 0.001). There is also a strong liner relation between the inverse of the stance duration and locomotion speed (*r* = 0.82, *p* < 0.001). No significant correlation between the swing duration and locomotion was found (*r* = 0.11, *p* = 0.34).

### Kinematics

#### Kinematics of the arms

**Figure 4:**
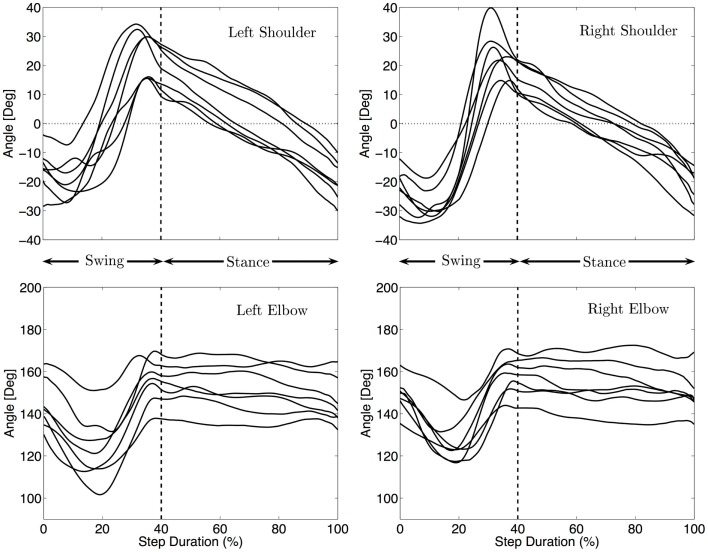
**Median fore limbs kinematics for each subject**. The trajectories were rescaled into normalized swing and stance phases, as explained in the Section “Materials and Methods”. The vertical dashed line indicates the touch-down of the hand. The same pattern of movement for all infants is visible: flexion/extension for the shoulder, elbow flexion, and extension during swing and little elbow yield during stance.

Figure 4 shows the median kinematics of the forelimbs for each infant. The shoulder is flexed at the beginning and extended at the end of the swing, before touch-down. The arm posture is quite protracted at touch-down, with joint angles between 14 and 39° for the different infants (Md = 24°). The shoulder during stance is mainly moving from the flexed posture to an extended one to allow the body to move forward. Lift off occurs at joint angles between −10 and −34°(Md = −26°). The movement of the limbs is qualitatively the same for every infant, only the total excursion angle changes. It ranges between 39 and 72° with a median total excursion angle of 46°.

The elbow joint is extended at the beginning and end of the swing phase while it is flexed at mid-swing. The total excursion during swing ranges between 16 and 45°(Md = 36°). During stance the elbow moves much less (3–15° among the infants, Md = 11°) and stays mostly at a quite extended position (median position between 135 and 170°, Md = 151°).

No correlation between the speed of locomotion and the amplitude of movement of the shoulders was found (correlation < 0.15 and *p* > 0.7).

#### Kinematics of the legs

**Figure 5:**
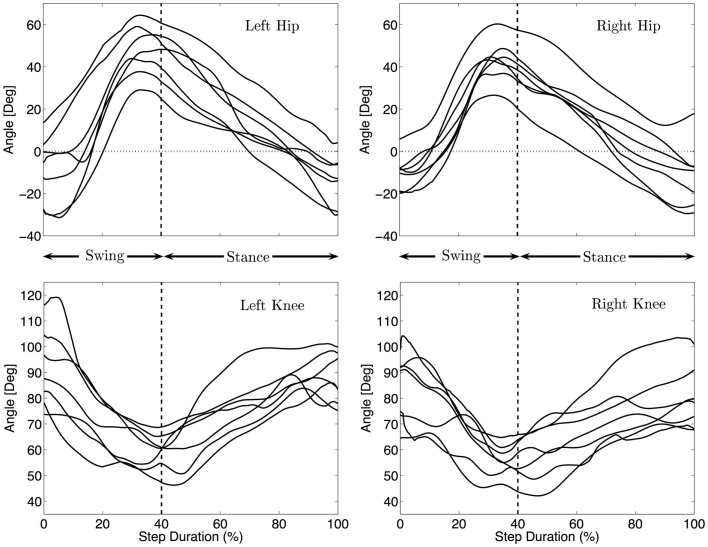
**Median hind limbs kinematics for each subject**. The trajectories were rescaled into normalized swing and stance phases, as explained in the Section “Materials and Methods”. The vertical dashed line indicates the touch-down of the knee. The same pattern of movement for all infants is visible: flexion/extension of the hip and flexion/extension of the knee.

Figure 5 shows the median angular values of the legs for each infant. During swing, the hip joint mainly flexes with a slight extension before touch-down of the knee, at touch-down the hip is very much protracted with an angle between 26 and 64° (Md = 44°). During stance the hip is extended, with an angle at lift off between −31 and 5° (Md = −11°). This behavior is qualitatively similar to the movement of the shoulder joints (in the sense of flexion and extension patterns) although the extension before touch-down is less visible as compared to the arm movement. Qualitatively, the movements of the legs are similar for all the infants, except that the amplitudes are different. The total excursion ranges from 52° to 75° (Md 57°).

The knee is always on the ground during stance where it is mainly used as a pivot around which the hip rotates. The median amplitude of the movement of the knee is 35°. Figure 5 shows that the knee flexes during swing and extend during stance mainly to follow the movement of the hips.

As for the arms, no significant correlation between the amplitude of the hips and locomotion speed was found (correlation < 0.25 and *p*-values > 0.5).

#### Kinematics of the spine

**Figure 6:**
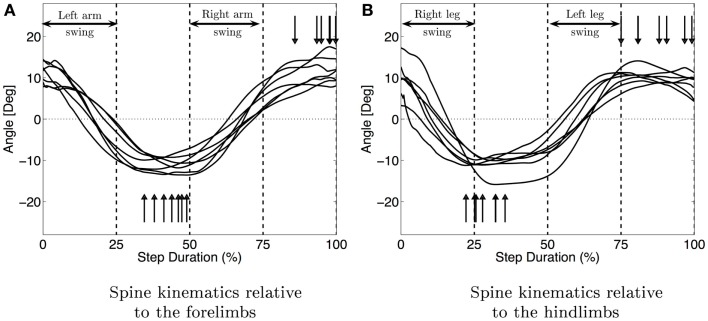
**Median movement of the spine for each subject**. **(A)** Shows the spine movement relatively to the fore limbs and figure **(B)** relatively to the hind limbs. The vertical arrows show the maximum curvature of the spine. The vertical bars delimit the stance and swing phases. The synchronization between spine movements and the limbs is clearly visible (movement of the spine during the swing of the arms and the maximum curvature during the stance).

Figure 6 shows the median movement of the spine in the horizontal plane for each infant. It also shows the coordination between the limbs and the spine. During the swing phase of the left arm and right leg, the spine is moving from a positive angle to a negative one (same values in magnitude) and is doing the opposite movement during the swing of the right arm and left leg. When the four limbs are on the ground, the spine is almost stationary. The spine movement is an oscillation synchronized with the swing phase of the limbs. The maximum curvature of the spine is attained for all the infants during the stance phase of the arms, which also corresponds for most infants but one to the stance of the opposite leg (see vertical arrows in Figure 6). The median amplitude of this movement is 23° with interquartile range of 9°.

**Figure 7:**
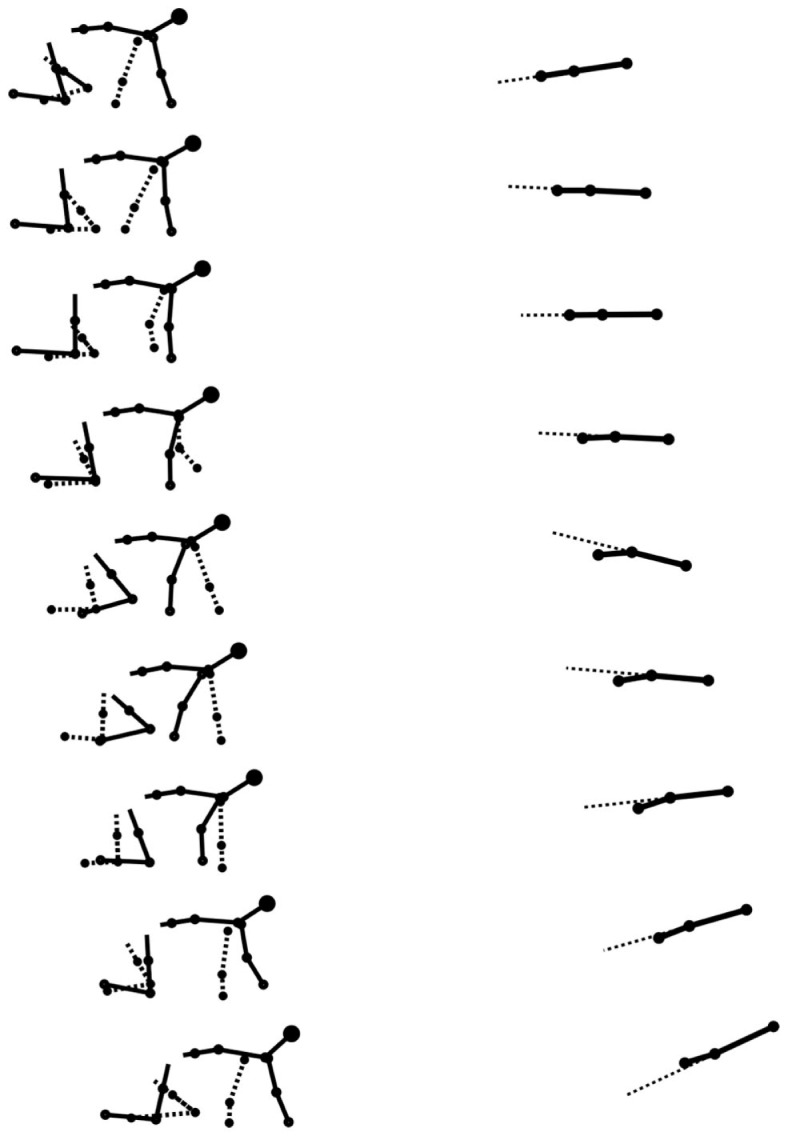
**Stick figures from real data of a typical crawling sequence (100 ms apart)**. The left graph shows the lateral view of the infant, the dashed line representing the left limbs. The right graph shows the spine movement of the same sequence from a top view, the dashed line here is an artificial extension of the front segment of the spine to help to see the curvature of the spine. The maximum curvature during the stance of the arms is visible.

Stick figures of a typical crawling sequence of an infant can be seen in Figure 7. This shows both the typical kinematics and limb coordination pattern of the crawling sequence.

### Crawling experience

**Figure 8:**
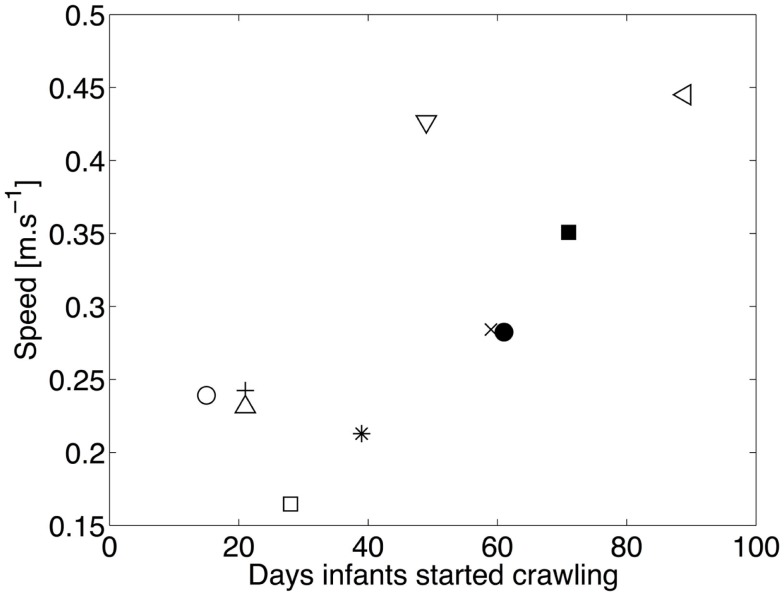
**Median speed of locomotion as a function of the number of days of crawling**. □ and ■ represent infant A for experiment 1 and 2, respectively. ◇ and ♦ are for B, ○ and ● for C, × for D, ⋆ for E, ◃ for F, and + for G. The linear correlation between the experience in crawling and the speed of locomotion is clearly visible.

Figure 8 shows the average speed of locomotion as a function of the number of days since the onset of crawling. The correlation between experience and speed of locomotion is high (*r* = 0.71, *p* = 0.021), indicating that the speed of locomotion increases with experience. Consequently, changes in the duration of stance are expected, as we pointed its relation with speed in the previous section.

**Figure 9:**
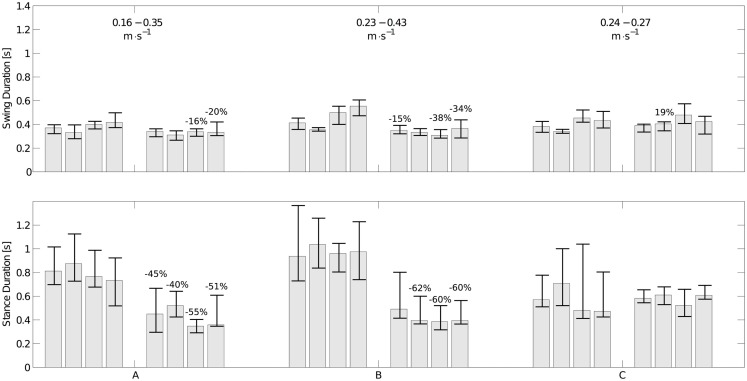
**Changes in swing (top figure) and stance durations (bottom figure) for the three infants measured twice**. The left set of bars corresponds to the first experiment; the right one corresponds to the second one. Each group of four bars represents the median durations of the left arm, the right arm, and the left and right legs, respectively. The interquartile range is represented as an error bar for each subject. The numbers on top of the bars represent the variation of median duration (only statistically significant variations are shown). The average locomotion speed in the 1st and 2nd experiment for each infant is shown in the top figure. The strong decrease in stance durations together with the increase in speed is noticeable for infant A and B, while there is neither a significant change in stance duration nor a significant change in speed for infant C.

Three of the infants were seen twice with approximately a month interval (see Table 1). The variations of the durations of stance and swing and the speed of locomotion are shown in Figure 9. Infants A and B show a significant decrease in stance and swing durations. The most important change is the stance duration, which is more than twice the variation of the swing duration. As expected a major increase of speed is also observed. Infant C did not show significant changes in swing and stance duration and his speed did not change much either. Limb kinematics in the second session is not significantly different from that in the first, suggesting that experience improves timing parameters rather than kinematics.

## Discussion

### Limitations and strengths of the study

The main limitation of this study is the small number of infants analyzed. However, the results are consistent among all infants. They are also consistent with previous findings on the crawling gait of infants. Another limitation is the restriction of the study to one gait while infants can adopt different crawling strategies. Therefore, the results cannot generalize to every crawling behavior, limiting the conclusions related to general principles from the developmental perspective.

The study has several strengths. To the best of our knowledge, it is the first quantitative study that combines both kinematics and gait/timing information for crawling infants and that includes the study of the spine movement in the horizontal place. Data collection was quite precise with a high bandwidth as 3-dimensional joint motions are collected at a 240 Hz rate.

### Main gait parameters related to speed

The standard crawling gait is between a walking trot and a lateral-sequence diagonal couplets walk, with the legs starting to swing shortly after the arms, which is in accordance with previous studies (7, 18, 26). As pointed out by these authors, infant crawling is different from other quadruped locomotion in terms of the discrepancy between the fore and hind limbs duty factor, which our data confirms. Stance duration varies considerably both for each infant and across infants and the variation is strongly correlated with locomotion speed. On the other hand, neither swing duration nor limb excursion appear to be correlated with locomotion speed. Therefore, our data suggest that the main strategy to change speed is to vary the stance duration. The relations between stance duration and speed seems generic to quadrupeds independently of their functional kinematics. For example, [Ref. (31), Chap. 4] showed through the physical simulation of quadruped models (robots) with different kinematic structures, including an infant like structure, which controlling the stance duration could always control locomotion speed, while swing duration had little influence on that. For other quadruped mammals, as speed increases, stance duration decreases in a similar manner (2, 5). The duration of the swing phase is relatively constant for all speeds (2, 3, 5). Thus from that point of view the temporal characteristics of crawling locomotion are the same as for other quadruped vertebrates.

### Kinematics

The general pattern of locomotion is qualitatively similar for all infants, which was expected since we chose to study the standard crawling gait (alternated locomotion on hands and knees); for infants having other gaits, there will certainly be differences in the kinematics. The standard gait in infants as well as in most quadruped mammals consists of a single period of protraction (during stance) and retraction (during swing), with beginning of protraction at the end of swing for the most proximal joints (shoulder and hip) (1, 2, 5).

At touch-down, the quite protracted arm posture [shoulder joint angle between 14 and 39° relative to the vertical (Md 24°)] is also typical of primates (13), whereas other mammals have a more retracted posture where the shoulder joint angle is negative. At lift off, the shoulder joint is not very much retracted (Md −26°) while most species show angles lower than −50°. In addition, the total shoulder excursion angle is relatively small (Md 46°). However, when looking in details at primate species, infants shoulder characteristics are quite close to the primates with the largest average body size, the Pongidae (i.e., great apes), where mean touch-down, lift off, and total excursion shoulder angles are 21, −33, and 55°, respectively (13). Larson et al. (29) published an extensive comparison on the hindlimb excursion angles of different mammals (including primates). Primates and marsupials show relatively high hindlimb angles at touch-down (33 and 40° resp.) as compared to other species (<27°). While at lift off the studied species all show angles lower than −17°, the highest being for primates (−29°). The total excursion angles are high for primates and marsupials (64 and 58° resp.) compared to other mammals (<52°). Compared to these data, infants have hindlimb angles at touch-down that are relatively high (Md 44°) and small retraction angles at lift off (Md -11°). The total excursion angle is high (Md 57°) and compares well with what is observed in other primates of the same weight. Finally, the hindlimb excursion range is much higher than that of the shoulder for infants, which is something that is also observed in Pongidae and koalas but not in other mammals (29). From that perspective, infants have a general limb kinematics that is really close to great apes.

The elbow joint performs a single flexion and extension during swing, which shortens the length of the arm to allow the limb to move forward, and remains straight during stance. The swing part corresponds well to the movement of the elbow joints of other mammals. The stance part is different and we observe a quite extended forelimb as compared to other mammals (32) and an elbow movement of very small amplitude. It is in contrast to other mammals where a flexion/extension of the limb is generally observed during this phase, and especially to primates who use a relatively compliant gait with a quite important elbow yield (14). Compliant gaits are generally characterized by a large elbow yield during stance, longer step length, and longer contact times. They involve an increase in metabolic power (33) that implies an increased effort of the muscles of the joints. The crawling gait is not compliant in contrast to other primates. However, the compliant gait of primates is tightly connected to arboreal locomotion and primates seem to use less compliant gaits on ground than on branches (14). The extended forelimb during stance is also characteristic of large animals (including primates) with relatively weak limb bones since such a posture reduces bone stresses (34). One might speculate that infants choose a gait that reduces bone stresses and metabolic power.

During standard crawling, the forelimb is quite extended and consists of two functional segments and the hindlimb of only one functional segment making the length of the forelimb (distance between the shoulder and the hand) greater than that of the hindlimbs. This difference in limb length can explain the differences in the excursion angles of the fore and hindlimbs (29), perhaps to keep a similar step length between the longer and shorter limbs. This limb difference could also explain the quite protracted fore limb at touch-down, since a retracted limb would lead either to an unstable gait if the elbow did not yield (since the center of mass would go out of the support polygon) or to the reduction of the visual field if the elbow yielded.

### Lateral-sequence footfalls

The footfall sequence during quadruped walking in primates is generally a diagonal sequence gait [left-hind (Lh), right-front (Rf), right-hind (Rh), and left-front (Lf)] while other non-primate quadrupeds use a lateral-sequence gait (LhLfRhRf sequence) (5, 6, 7). Despite some primates and especially their infants choosing a lateral-sequence gait (10), the diagonal sequence gait is the dominant one (11). The crawling gait of infants is thus closer to non-primate quadrupeds, since infants only use a lateral-sequence gait. This result is consistent with previous findings on crawling infants (7, 18, 26). We must note that this lateral-sequence gait is not merely characteristic of human infants but also of adults as reported by Patrick et al. (26).

The gait chosen by infants is the most stable pattern of coordination possible. The lateral-sequence gait (non-primate) is the only pattern of coordination where the projection of the center of mass on the ground stays in the support polygon^2^ when at least three feet are on the ground (35). When at least two limbs are on the ground, this pattern of coordination minimizes the duration of phases where the projection of the center of mass is outside the support polygon and certainly increases stability.

A lateral-sequence gait close to a trot implies that the hand will start to swing just before the contralateral leg, giving some precedence to the swing of the hand. During the experiments, infants were spontaneously starting to crawl toward a goal or an object with the hand directed toward the object as if they were trying to reach it. Adolph et al. (17) also mention that infants initiate locomotion with the hands. Since infants reach for and manipulate objects all the time with their hands and since the fore limbs are the only visible limbs, it could also explain the preference of the fore limbs for the start of swing, i.e., the start of locomotion. That may indicate a visual-motor coupling with the purpose to direct the infant to goals. Further experiments would be needed to confirm this hypothesis.

### Spine kinematics

The periodic lateral undulation of the spine is synchronized with the limbs, with maximum amplitude reached just after ipsilateral hind limb touch-down. The observation of a standing wave has already been made for many tetrapods, from salamander (36–38), lizards (39–41) to primates (Strepsirhines) (9). For strepsirhines, the maximum amplitude curvature of the spine is reached after ipsilateral hind limb touch-down and for the lizards it is reached before (except at very low speeds). As hypothesized by Shapiro et al. (9), the difference in the timing of the maximum curvature in lizards and these primates could come from their respective gait. Lizards use a lateral-sequence walking gait while primates use a diagonal sequence walking gait. However, infants have maximum curvature of the spine similar to primates but a different gait. As opposed to the strepsirhines, infants have long fore limbs when compared to hind limbs, which could explain these differences. If the spine movements and gait of infants were closer to slowly walking lizards than primates, an increase in their speed would lead to a maximum curvature happening before the end of swing. More experiments both on human and non-human primate infants using sometimes a lateral-sequence gait would be needed to further compare gaits and spine movements and explore further explanations. For example, one question that could be explored is whether infants use different mechanisms to adjust speed and posture, keeping posture by modifying the swing-spine dynamics on one hand and adjusting speed by varying stance duration on the other hand.

### Development of the gait through experience

It seems that, apart from stability, the main experience dependent parameter is the duration of stance phase. The swing phase remains rather constant with experience. The crawling speed is related to experience, and the crawling gait of infants becomes faster when the complete support phase (where the four limbs are on the ground) is shorter. It means that infants begin with a gait in between a walk and a walking trot and tend to a perfect walking trot with more experience. Interestingly, there are similarities with the development of the gaits of other quadrupeds. Indeed, in several rodent species lateral walking appears first closely followed by trotting, and more specialized gaits (asymmetric or biped gaits) develop only later (42).

## Conclusion

The similarity between the infant standard crawling gait and that of other mammals, especially non-human primates is described by four results. Firstly, a positive correlation between stance duration and speed, secondly, the protracted limb at touch-down, thirdly, the relatively extended arms during locomotion, and fourthly, the coordination of the spine with touch-down of the limbs. These similarities may be reflected by a common principle underlying neural control (43) or by the mechanical constraints arising during quadruped locomotion. However, the infant crawling gait differs from other primates in at least two aspects: infants use lateral-sequence footfalls, which make their pattern of coordination closer to other mammals, and a relatively stiff elbow during stance. These differences could be related to stability (for the gait) and to reducing joint efforts (for the stiffness).

## Conflict of Interest Statement

The authors declare that the research was conducted in the absence of any commercial or financial relationships that could be construed as a potential conflict of interest.

## Footnotes

^1^Diagonal couplets mean that the gait is closer to a trot than to a pace

^2^The support polygon is the area delimited by the limbs that are on the ground, for example when three limbs are on the ground this polygon is the triangle created by these limbs. In mechanics it is known that if the projection of the center of mass on the ground is located inside the support polygon then the body is (statically) stable under gravity otherwise the body is not stable and an action is required to keep the body balanced (accelerating the center of mass or taking a step for example). While this stability criterion is valid for bodies that do not move, it still remains very good indicator for bodies with low acceleration.
